# Temporal and Spatial Expression of Arabidopsis Gene Homologs Control Daylength Adaptation and Bulb Formation in Onion (*Allium cepa* L.)

**DOI:** 10.1038/s41598-019-51262-1

**Published:** 2019-10-10

**Authors:** Md. Harun Ar Rashid, Wei Cheng, Brian Thomas

**Affiliations:** 1 School of Life Sciences, Gibbet Hill Campus, The University of Warwick, Coventry, CV4 7AL UK; 2 School of Life Sciences, Gibbet Hill Campus, The University of Warwick, Coventry, CV4 7AL UK; 3 Present Address: Department of Horticulture, Bangladesh Agricultural University, Mymensingh, 2202 Bangladesh

**Keywords:** Environmental impact, Transcriptomics, Light responses, Gene expression, Plant molecular biology

## Abstract

Genetic studies aimed at onion improvement have been limited because of high heterozygosity, a very large genome size with a high level of repetitive DNA and a biennial life cycle. Onion bulb initiation is daylength-dependent, which places a significant barrier to adapting new varieties for growth at different latitudes. Compared to the photoperiodic regulation of flowering, relatively little is known about genetic regulation of the bulbing process. This study aims to identify the role of gene sequences involved in daylength-regulated bulb formation and tissue specific expression of onion. A comprehensive set of developmental and spatial quantitative mRNA expression experiments were carried out to investigate expression of onion *FLOWERING LOCUS T* (*AcFT*), *LEAFY* (*AcLFY*) and *GIBBERELLIN-3 OXIDASE* (*GA3ox1*) during the bulbing response. Bulbing ratios were used to measure the response of onion plants under long day (LD) and short day (SD) conditions. *AcFT1* was expressed in LD, which induces bulb formation, while *AcFT4* was expressed in SD, which inhibits bulb formation. *AcFT5* and *AcFT6* were expressed in LD and might also be involved in bulb formation itself. All *AcFT, AcLFY* and *GA3ox1* genes showed distinctive patterns of tissue specific expression in onion, with *AcFT* genes found primarily in the sites of perception in the leaf and LFY in the basal tissues, the site of response. The results are consistent with *AcFT1* expression being the signal for LD-induced bulb initiation and *AcFT4*, being involved in suppressing bulbing in SD.

## Introduction

Onion (*Allium cepa* L.) is an edible monocotyledonous bulbous perennial (often biennial) vegetable crop, belonging to the family *Alliaceae* and is cultivated in temperate, tropical and sub-tropical regions^1–3^. It is included in the order Asparagales, the second most economically important monocotyledons, next to Poales, which include the cereal crops^2,4^. Global production of onions in 2016 was 82.85 million tonnes from 4.2 million hectares of land. The Food and Agriculture Organization of the United Nations^5^ reported that in terms of total annual world production, onion ranks second among horticultural crops, next to tomato. Onion is consumed at both the green and mature stage for salad and spice. In addition to its economic importance, it has nutritional and health benefits because of its high content of valuable phytochemicals, antioxidants, and sulphur compounds, which contribute to its flavour.

The onion life cycle can be divided into three main stages, namely seedling, bulb and flowering^6,7^. In temperate onion, during the first growth phase onion seed will start to germinate after sowing^1^. After a few days of germination, the seedlings/sets are planted in the spring and the plants undergo a juvenile phase of growth during which plants will not bulb regardless of being exposed to inductive conditions. Following that, onion leaves require continuous exposure to a suitable photoperiod in order to initiate and complete bulbing^2^. Onion leaves are composed of a photosynthetic leaf blade and a fleshy leaf sheath/storage leaf base or scale^8^. After receiving the photoperiodic stimulus, bladed green leaves formation near the apical meristem ceases and they are transformed into only bladeless leaves. Bulb sheath cells expand in response to a photoperiod signal from the leaf blade and act as a sink for the leaf carbohydrates such as glucose, fructose, sucrose and fructans. With the maturity of the bulb, the outer (oldest) one to four leaf scales dry out and become protective skin^1^. The onion plant then overwinters as a bulb and during this time, if the environment is favourable, flowering is induced after vernalisation in response to prolonged cold temperature. The onion plant then flowers and sets seed during the spring/summer of its second year of growth and thus its life cycle is completed^1,9^.

Bulb formation in temperate onion is daylength-dependent and the leaves of the plant are the photoperiodic stimulus receptor^10^. Bulb initiation can be defined as the point at which the ‘bulbing ratio’, the ratio of the maximum bulb diameter at the base to the minimum at the neck/sheath, increases to greater than two (>2)^11^. Long-day (LD) onions are grown in temperate regions and require at least 14 or more hours of light to stimulate bulb initiation, while, short-day (SD) onions are grown in more tropical regions and require a daylength of only 10 h or more for bulbing^2^. The matter is further complicated as some onion varieties are intermediate where they need 12 h or more of daylight before they will start producing the onion bulb. This daylength-dependent bulb initiation is similar to the photoperiodic regulation of flowering in other plants^12,13^. Therefore, it is hypothesised that the genes involved in the daylength regulation of flowering in Arabidopsis are also responsible for the daylength regulation of bulb formation in onion. Both processes are induced by LD, signal perception is in the leaf blade, response at the meristem and both are promoted by far-red light, through phytochrome A (PHYA)^14,15^. Bulbing is a reversible process and plants grown under inductive conditions promote bulb formation but if transferred to non-inductive condition, they rapidly revert to vegetative growth^11,16^.

The flowering locus T gene (*FT*), first identified in *A. thaliana*^17,18^, has been shown to be the major component of the floral signal molecule, florigen^19^. This *FT* plays a major role in the photoperiodic pathway for the initiation of flowering in the apical meristem with the help of other floral homeotic genes like *LFY*^20^. Furthermore, *FT* is a target of constans (*CO*) and acts upstream of suppressor of constans overexpression (*SOC1*) and can act as a mobile flowering signal to induce flowering by long-distance transportation^21,22^. For bulbing, as with flowering, daylength perception occurs in the leaves, while the response is in the meristem^2^. These suggest that a mobile signal with properties similar to *FT* might be involved.

In addition to the regulation of flowering, *FT* genes have been found to be involved in a range of physiological processes, suggesting more general function as a plant hormone^9^. For example, an *FT* promotes vegetative growth and inhibition of bud set in poplar in response to warm temperatures and LD photoperiods^23–25^, in tomato and maize, *FT* genes have been found to function as general growth regulator^26,27^. Other than vegetative growth and flowering, *FT* is also involved in the SD induction of tuberisation in potato^28^.

Characterising genes involved in the daylength requirement of bulb formation will help in understanding the basis of the difference between different daylength types, which is important for adapting new varieties for growth and development at different latitudes. Six *FT* genes (*AcFT1-6*) have been identified in double haploid onion line CUDH2150 and the authors proposed that *AcFT1* and *AcFT4* genes promote and inhibit bulb formation, respectively, while *AcFT2* acts to promote flowering15. In addition, *FKF1* and *GI*, which are involved in the photoperiodic regulation of flowering in Arabidopsis are also conserved in onion^12^.

This study characterises the developmental and spatial expression of putative photoperiodism-related genes by quantitative gene expression analysis in different response types of onion under a range of bulbing and non-bulbing conditions in order to further understand their potential roles in the daylength regulation of bulbing.

## Materials and Methods

### Plant materials

The long day (LD) onion (*Allium cepa* L.) variety ‘*Renate F1*’ (also called *Renate*) (Elsoms Seeds Ltd., Spalding, UK) and the short day (SD) onion variety ‘*Hojem*’ were used for these experiments. Varieties with different daylength responses were originally sourced from the Warwick Genetic Resources Unit and the seeds provided by Dr. Andrew Taylor, Warwick Crop Centre. Seeds of *Hojem* were collected from the Vegetable Genetic Improvement Network (VeGIN, UK) project Diversity Set.

### Time-course experiment to study gene expression for Renate F1 during development

#### Experiment 1

This experiment was conducted to characterise the response of bulb initiation in relation to daylength as a prelude to more detailed later experiments. Onion plants were grown in natural conditions within a glasshouse at the Crop Centre in Wellesbourne during the period from 6^th^ March to 7^th^ May 2013 when daylight was 11 h 15 min to 15 h 37 min, respectively. Initially, *Renate F1* seeds were sown in modular trays and after 4 weeks plants were potted up into 9 cm pots containing Levington M2 compost (Supplementary Fig. S1). At 48 d when plants had initiated bulbing, half of them were transferred to constant LD (16 h photoperiod including 8 h fluorescent followed by 8 h incandescent light) and the other half to constant SD (8 h fluorescent light) in SANYO 2279 controlled environment cabinets at the Phytobiology Facility for another 2 weeks (Supplementary Fig. S2). Both SANYO cabinets were set at 22 °C day and 18 °C night temperatures with 60% relative humidity and ambient CO_2_ concentration, and provided with a Photosynthetic Photon Flux Density (PPFD) of 100 Wm^−2^. Sampling was carried at 62 d at Zeitgeber time 10 (ZT10) and involved removing the middle part of the first newly expanded leaf and the middle to the basal portion of bulb, chopped into small pieces, and freezing in liquid nitrogen before storing at −80 °C. The harvested materials were used for molecular analyses.

#### Experiment 2: Generating materials for molecular analyses

Onion plants were grown in natural conditions in a glasshouse at the University of Warwick Phytobiology Facility but otherwise as described for experiment 1. They were grown during the period from 19^th^ June to 6^th^ August 2013 when daylight was 16 h 38 min to 15 h 7 min, respectively. At 48 days from sowing (DFS), the rest of the plants were divided into 3 groups, one group was transferred to constant LD, one to constant SD, using similar controlled condition as described in Experiment 1, and one group kept in NC. From 30 d, measurements of bulb and neck diameter were taken using slide callipers at weekly interval for 2 weeks and then twice a week for the rest of the growth period. Onion leaf and bulb material was harvested at ZT10 and at sampling, 3 plants were pooled together for replication using a Completely Randomised Design (CRD). Sampling removing the middle part of the first newly expanded leaf and the middle of the white basal portion of the leaf chopped into small pieces, and freezing in liquid nitrogen before storing at −80 °C. Plants were selected for harvesting using a random number generator^29^. The harvested materials were used for molecular analyses. ‘Bulbing ratio’ was calculated by dividing the maximum bulb diameter by the minimum neck diameter and bulb initiation was considered to have been initiated when the bulbing ratio reached a value greater than 2^30^. Means, standard deviations and standard errors were calculated for all data points using Microsoft Excel and the significance of the differences in bulbing ratio between treatments were assessed by using factorial analysis of variance (ANOVA) with repeated measures. ANOVA was carried out using statistical software package SPSS.

### Time-course experiment to study gene expression for Hojem during development

The plants were grown during the period from 17^th^ March to 8^th^ August 2014 in a photoperiod-controlled glasshouse compartment of the Phytobiology Facility to give 12 h daylight and provide other environmental conditions as for *Renate*. From 35 d, measurements of bulb and neck diameter were taken from both varieties at weekly interval throughout the growth period using slide callipers. Harvesting and sampling was carried out and stored at −80 °C. Plants were selected for harvesting using a random number generator^29^. The harvested materials were used for molecular analyses. ‘Bulbing ratio’ was calculated and statistical analysis was conducted.

### Spatial patterns of gene expression in leaves of Renate F1

Onion plants were grown under NC in the glasshouse at the University of Warwick Phytobiology Facility during the period from 26^th^ July to 27^th^ September 2013 when daylight was 15 h 42 min to 11 h 52 min, respectively. Supplementary illumination with HPS lamps was provided to maintain a minimum 16 h daylength. At 61 d when plants had initiated bulbing, half of them were transferred to constant LD (16 h photoperiod including 8 h fluorescent followed by 8 h incandescent light) and the other half to constant SD (8 h fluorescent light) in SANYO 2279 controlled environment cabinets at the Phytobiology Facility for another 2 weeks. On the last day, plants were harvested at ZT10 and leaves were separated. The 5^th^ number leaf was taken and cut into 12 pieces of 1 cm starting from the base and 6 plants were pooled together for both LD and SD conditions (Supplementary Fig. S3). The samples were immediately frozen in liquid nitrogen before storing at −80 °C. The harvested materials were used for molecular analyses.

### Gene identification and isolation

Key genes, which have known functions in Arabidopsis flowering and regulate other important pathways such as sucrose and gibberellins pathways, were selected. The sequences of each gene in Arabidopsis were obtained from NCBI database (www.ncbi.nlm.nih.gov). An onion EST sequence was obtained by blasting the sequence of each gene in Arabidopsis homologs against onion database (www.ncbi.nlm.nih.gov/nucest/?term=onion). After obtaining the EST sequences, they were aligned with Arabidopsis sequences using MegAlign^TM^. ESTs were then used to design primers (Supplementary Table S1). From alignment information, the positions of sequence identity were obtained and primers (Forward and Reverse) for each gene amplification designed using Primer3Plus (http://www.bioinformatics.nl/cgi-bin/primer3plus/primer3plus.cgi.

### RNA extraction, DNase treatment, cDNA synthesis and sequencing of PCR products

Total RNA was extracted from leaf and bulb material from onion grown under LD and SD using the Z6 buffer method, following the manufacturer’s (Roche manufacturing Ltd., Republic of Ireland) guidelines. Samples were ground using pestle and mortar and then approximately 100 mg of frozen plant tissue was homogenised using a Dremel drill. In this step, Z6 buffer reagent and b-Mercaptoethanol were added which act to remove RNase. Two extra reagents, 3 M Sodium acetate (NaOAC) and 7.5 M Lithium chloride, which remove carbohydrates and polysaccharides, respectively, were included in this method to obtain high quality RNA. After isolation, the quality and quantity of total RNA was measured with the Thermo Scientific NanoDropTM 1000 Spectrophotometer (NanoDrop Technologies, Inc., USA).

The TURBO DNA-free treatment kit (Ambion, USA) was used to eliminate the genomic DNA contamination following the manufacturer’s guidelines. A PCR was set up to check for genomic DNA contamination using primers for ALLINASE (ALL) gene and visualized on RNA gel electrophoresis. Sequencing of PCR products from genomic DNA confirmed that the primers contained no mismatches. cDNA was synthesised using 2 μg total RNA using ThermoScriptTM Reverse transcription polymerase chain reaction (RT-PCR) System (Invitrogen by Life Technologies, Cat. No. 11146-016) for RT-PCR using oligo(dT) following the manufacturer’s guidelines and subsequently treated with RNase H. PCR products were purified following PCR and agarose gel electrophoresis using QIAquick PCR Purification Kit (QIAGEN) and QIAquick Gel Extraction Kit (QIAGEN), respectively, following the manufacturer’s guidelines and samples were eluted in 30–50 µl of SDW. For gel purification, bands were cut out under UV light with a wavelength of 302 nm (Bio-Rad UV Transilluminator 2000) using a scalpel blade. A volume of 1 µl purified DNA was quantified using a NanoDrop™ ND-1000 spectrophotometer (Thermo Scientific). A total amount of 10 μl (Premix 5 μl template of 20–80 ng/μl conc. + 5 μl Primer of 5 pmol/μl conc.) purified PCR products were sent to GATC Biotech for sequencing. Sequence files were viewed and edited using the EditSeq package of DNAStar Lasergene (DNAStar Inc.). Chromatograms where analysed and interpreted using 4Peaks Chromatogram and edited using SeqMan^TM^, SeqBuilder^TM^ and MegAlign^TM^ of DNAStar Lasergene (DNAStar Inc.).

### Analysis of gene expression using qRT-PCR

Total RNA was extracted from 100 mg of leaf material harvested at each time point using Trizol^®^ reagent (Invitrogen) following the manufacturer’s guidelines. Samples were DNase treated using TURBO DNA-freeTM (Ambion) and first-strand cDNA synthesized from 2 μg of total RNA using SuperscriptTM II Reverse Transcriptase (Invitrogen) following the manufacturer’s guidelines^12^. The expression of reference genes and genes of interest was analysed by qRT-PCR using the CFX384 Touch^TM^ Real-time PCR machine from BioRad (Bio-Rad Laboratories Ltd. UK). For all other qRT–PCR analyses, a MESA GREEN qPCR MasterMix for SYBR^®^ green with fluorescein (Eurogentec) was used, following the manufacturer’s guidelines. Reactions were carried out in 15 μl volumes, containing 0.5 μl of cDNA. Three replications (triplicate) were carried out for each sample and the average CT value calculated. The protocol and primer details are provided in Supplementary Tables S2–S5. The qRT-PCR data were normalised against the house keeping genes *PP2AA3*, *PP2A1, TIP41* and *UBL* for each sample (Supplementary Table S3) using Biogazelle qBase+ software (www.biogazelle.com). The same housekeeping genes namely *PP2A1*, *UBL* and *TIP41* were used in all the developmental time course experiments and the housekeeping genes viz. *PP2AA3*, *UBL* and *TIP41* were used in all the spatial time course experiments. The significance of the differences in gene expression between treatments were assessed by using two-way analysis of variance (ANOVA). ANOVA was carried out using statistical software package Prism 7.

## Results

### Daylength regulation of bulbing in onion

**Figure 1:**
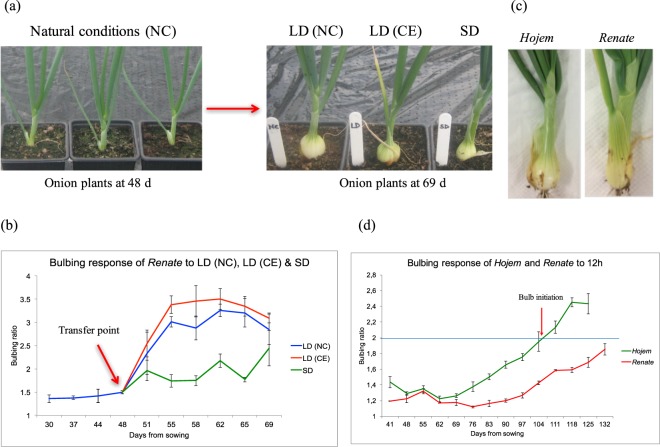
Bulbing response in onion grown under different daylengths at different stages of development. (**a**) Comparison of *Renate* plants grown under different daylengths at different stages of development. The left panel shows plants at the time of transfer at 48 DFS. The right panel shows plants from different treatments at 21 days after transfer (DAT). (**b**) Bulbing response of *Renate* plants grown under different daylengths. Error bars represent the SEM. Three plants were used in each data point. (**c**) Comparison of *Hojem* and *Renate* at 104 d under 12 h daylight. (**d**) Bulbing response of *Hojem* and *Renate* plants grown at 12 h daylight. Error bars represents the SEM.

Plants were initially grown under natural conditions, transferred to LD or SD in controlled environment (CE) at 48 d or kept under natural LD conditions (NC). A clear visible difference was observed in bulb development between plants grown at different daylengths (Fig. 1a). At 21 days after transfer (DAT), the LD (NC) and LD (CE) plants showed increased bulb diameter, which consequently increased bulbing ratio (Fig. 1b), whereas, the plants in SD showed only a small increase in bulb diameter, and continued producing new leaves, which resulted in an increase in neck diameter. Samples from this experiment were used for gene expression analysis. From ANOVA results, it was found that LD and SD treatments were significantly different from each other and the number of days from sowing had a significant effect on bulbing ratio (Supplementary Table S6). Moreover, the interaction between days from sowing (DFS) and daylength was shown to be significant showing that the pattern of bulbing ratio during the period of development of onion is affected by daylength. Therefore, it was confirmed that bulb initiation in *Renate* was controlled by daylength with LD conditions stimulating the bulbing process^16^.

To compare the responses of the LD variety *Renate* with the SD variety *Hojem*, plants were grown in a photoperiod-controlled glasshouse compartment of PBF at 12 h daylight. A clear difference was observed in bulb development between *Hojem* and *Renate* plants (Fig. 1c). Statistical analysis also showed that bulbing ratios were significantly different between *Hojem* and *Renate F1* varieties in response to the daylength (Supplementary Table S7). Taking a bulbing ratio of 2 as representing bulb formation, bulbing was observed in *Hojem* at around 104 DFS, but was not seen in *Renate* even at 132 DFS (Fig. 1d). This reflects the critical daylength requirement, where SD onions start making bulbs at 10 to 12 h of daylight, while temperate (LD) onion requires at least 14 h of daylight to stimulate bulbing process^13^. Even though 12 h daylength would be long enough for bulbing in SD varieties of onion, there was a long period (104 DFS) to bulb initiation in *Hojem*, compared to the time to bulbing in *Renate* plants grown initially in NC. This could be due to the low light integral during the early stage of plant growth in the 12 h daylength chamber compared to NC^31^. Samples from this experiment were used for gene expression analysis.

### Gene identification and isolation

In preliminary RT-PCR experiments, the expression of the genes under study was determined in pooled samples of *Renate F1* using qRT-PCR primers (Table 1). PCR products were run on a gel and showed a clear band at the right product size (Supplementary Fig. S4). All were shown to represent the expected gene through sequencing of PCR products. *FT3* shared 83% identity with *FT5*, and these two genes were not distinguished in the qRT-PCR analysis. In the initial PCR, *AcFT1* and *AcFT4* mRNA bands of *Renate F1* pooled sample were not clearly visible on the gel (Supplementary Fig. S4a), suggesting they were expressed at a relatively low level. However, re-amplified PCR showed clearly visible bands on the gel (Supplementary Fig. S4b). In addition, clear cDNA bands for *Hojem* and *Renate F1* grown under 12 h daylength were also found on gel (Supplementary Fig. S4c–f).

**Table 1:** Primers for qRT-PCR used to estimate the expression of genes of interest in onion. Forward is top in each section and Reverse is below.

Gene	NCBI GeneBank Accession	Forward (qRT-FOR) and Reverse (qRT-REV) primer sequences (5′…..3′)	Annealing temperature (°C)	Product size (bp)	Primer concentration for qPCR (µM)
*AcFT1* (726 bp)	KC485348	AAACCATCACAAATAACTCAGCA GTTTCTCGCCCAAAGTTCG	56	185	0.2
*AcFT2* (572 bp)	KC485349	AAGTTGCTAATGGACGCGAGTTTAAG CACCAACACAAGTGCATAAGAGTTCC	61	104	0.2
*AcFT3* (745 bp)	KC485350	AGGAAGTTACTAACGGGTGTGAA CAAAGCTTGCATCTTTTGACC	60	201	0.2
*AcFT4* (539 bp)	KC485351	TGAAATAGGAGGTGTACCAAGAAT TTCCGAAACTACCATCCATATTTG	60	143	0.3
*AcFT5* (604 bp)	KC485352	GAAATTGGAGGACGCGAC CTTGCATCTTTTGCTTCTGGTA	60	137	0.2
*AcFT6* (841 bp)	KC485353	TCGTCAATCGATGGTTATAAATCA TTTCCATAACTTGCATCGACTGT	60	180	0.2
*AcLFY* (1119 bp)	JX275963	AGCGTGCTATCAACCGATAGTAGTGA AGCTTAGTCGGAACATACCAAATGGA	60	108	0.3
*GA3ox1* (1208 bp)	AB303422	GCTATTTGACAAAGCCCTAGCATCTG ATCATACGCAACTAAGCAAGCATGTG	63	86	0.3

Legends: *AcFT: A. cepa FLOWERING LOCUS T, AcLFY: A. cepa LEAFY, GA3ox1: GIBBERELLIN 3-OXIDASE 1*.

### Temporal gene expression in onion in response to LD and SD

#### Expression of FT genes in different daylengths

**Figure 2:**
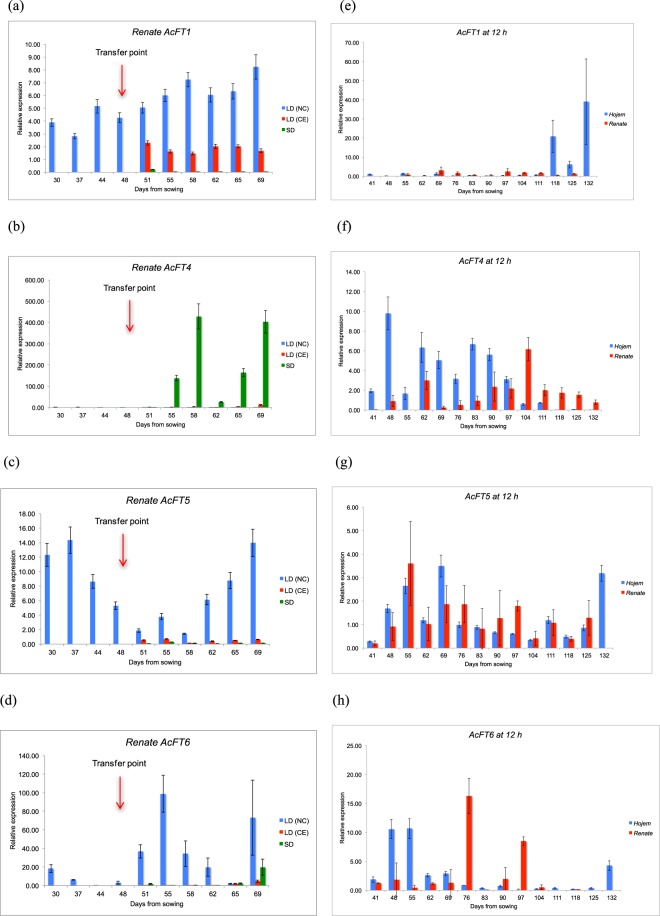
Expression of *FT* genes in *Renate* leaf tissue at different daylengths. Error bars represent the SEM. (**a**) Expression of *AcFT1* in *Renate* leaf tissue at different daylengths. *AcFT1* was expressed in LD (NC) and LD(CE) but not expressed in SD. (**b**) Expression of *AcFT4* in *Renate* leaf tissue at different daylengths. *AcFT4* was only expressed in SD but was not expressed in LD (NC) and LD (CE). (**c**) Expression of *AcFT5* in *Renate* leaf tissue at different daylengths. *AcFT6* was expressed in LD (NC) and LD (CE) but not in SD. (**d**) Expression of *AcFT6* in *Renate* leaf tissue at different daylengths. *AcFT6* was expressed in LD (NC) and LD (CE) but not in SD. (**e**) Expression of *AcFT1* in *Hojem* and *Renate F1* leaf tissue at 12 h daylight. *AcFT1* was expressed in *Hojem* during bulb formation and development, while, showed very limited expression in *Renate*. (**f**) Expression of *AcFT4* in *Hojem* and *Renate F1* leaf tissue at 12 h daylight. *AcFT4* was expressed in *Hojem* at the early stage of growth before bulb formation and was expressed in *Renate* at the later stage of growth and development. (**g**) Expression of *AcFT5* in *Hojem* and *Renate F1* leaf tissue at 12 h. *AcFT5* was expressed in both *Hojem* and *Renate* throughout the development. (**h**) Expression of *AcFT6* in *Hojem* and *Renate F1* leaf tissue at 12 h daylight. *AcFT6* was expressed in both *Hojem* and *Renate* at the early stage of the development.

In *Renate*, *AcFT1* was expressed in LD (NC) and LD (CE), i.e. the conditions that promoted bulbing, but not expressed in SD, where foliage leaves continued to be produced (Fig. 2a). It was observed that the level of expression of *AcFT1* was significantly higher in LD (NC) than to LD (CE). Two-way ANOVA confirmed that the difference in *AcFT1* expression between LD (NC), LD (CE) and SD conditions was significant (Supplementary Table S7). This result is consistent with the earlier study conducted by Lee *et al*. (2013), where the authors found that *AcFT1* was only expressed in LD-grown plants but was not expressed in SD-grown plants. *AcFT4*, in contrast, was expressed in SD but was not expressed in LD (NC) or LD (CE) conditions (Fig. 2b). This result is also consistent with the previous study conducted by Lee *et al*. (2013), where they proposed that *AcFT4* prevents *AcFT1* upregulation and inhibits bulbing in transgenic onions^9^. Therefore, it could be confirmed that the expression of *AcFT4* is negatively correlated with *AcFT1*.

It was also seen that *AcFT4* expression was not detected during the early stages of seedling growth, even though the natural daylength was less than the critical daylength of 14 h for LD varieties. Two-way ANOVA confirmed that the difference in *AcFT4* expression between LD (NC), LD (CE) and SD conditions was significant (Supplementary Table S8). It was also confirmed that the number of days from sowing had a significant effect on *AcFT4* expression (Supplementary Table S9). This suggests that *AcFT4* is under developmental as well as daylength regulation.

*AcFT5* was strongly expressed throughout the development in LD (NC), but showed very limited expression in LD (CE) and it was not expressed in SD (Fig. 2c). The level of expression was significantly higher in LD (NC) than to LD (CE). Comparing different daylengths, the expression of *AcFT5* is consistent with *AcFT1*, however, *AcFT5* showed an interesting pattern of expression, which is different to that of *AcFT1*. It was observed that *AcFT5* showed higher expression during early stage of growth and the expression was sharply decreased at the time of bulb formation and then sharply increaed during the rest of the bulb development period. However, this result is inconsistent with the previous study conducted by Lee *et al*. (2013), where the authors proposed that *AcFT5* expression did not appeared to be strongly affected by daylengths^9^. ANOVA confirmed that the difference in *AcFT5* expression between LD (NC), LD (CE) and SD conditions was significant. It was also confirmed that the number of days from sowing had a significant effect on *AcFT5* expression (Supplementary Table S10).

*AcFT6* was only expressed in LD (NC) but not in LD (CE) or SD conditions (Fig. 2d). In addition, the expression level of *AcFT6* in LD (NC) was very low during the early stage of plant growth and was only higher immediately after bulb initiation. A limited expression of *AcFT6* was found in both LD (CE) and SD conditions at the later stage of bulb development in *Renate*. The difference in *AcFT6* expression between LD (NC), LD (CE) and SD conditions was statistically significant. It was also confirmed that the number of days from sowing had a significant effect on *AcFT6* expression (Supplementary Table S11).

### Expression of FT in 12 h days

In *Hojem* at 12 h, bulbing took place at about 104 DFS and became detectable at 118 DFS (Fig. 1c). *AcFT1* showed higher levels of expression during the later stage of bulb development and maturity (Fig. 2e). The data suggest that *AcFT1* induces bulb formation and development in *Hojem*. On the other hand, bulbing was not observed in *Renate* at 12 h daylength even at 132 DFS and *AcFT1* showed very limited expression throughout the development period. This result is consistent with the results in the previous experiment, where *AcFT1* was not expressed in *Renate* at 8 h daylength. Thus, *AcFT1* is only expressed when the daylength is greater than 12 h in *Renate*, which is consistent with it having a role in the daylength dependence of bulbing. Two-way ANOVA confirmed that the difference in *AcFT1* expression between *Hojem* and *Renate F1* under 12 h daylength was significant. It was also confirmed that the number of days from sowing had a significant affect on *AcFT1* expression (Supplementary Table S12).

In *Hojem* at 12 h, *AcFT4* showed higher expression at the early stage of plant growth and was down-regulated at about the time of bulb formation (Fig. 2f). Lee *et al*. (2013), found that *AcFT4* was expressed at relatively high levels in leaves of young seedlings under both SD and LD contions^9^. However, in *Renate* at 12 h, *AcFT4* overall expression was low compared to experiment 1. *AcFT4* was expressed during the later part of development and expression was up-regulated at the time when plants would generally bullb if in LD. The difference in *AcFT4* expression between *Hojem* and *Renate F1* under 12 h daylength was statistically significant (Supplementary Table S14).

*AcFT5* was expressed throughout the growth and development period in both *Hojem* and *Renate* at 12 h daylight (Fig. 2g) with no significant difference found between two varieties. (Supplementary Table S15). In contrast, *AcFT6* was expressed at the early stage of plant growth in *Hojem*, while it was expressed at the middle stage of development in *Renate*. (Fig. 2h). *AcFT6* was down-regulated before bulb formation in *Hojem* and was not expressed during the rest of the bulb development. The interaction between days from sowing and variety was also shown to be statistically significant (Supplementary Table S16), showing that the expression of the *AcFT6* over time is affected by daylength.

The expression of *AcFT2* was also assessed in the 12 h experiment. Lee *et al*. (2013) proposed that FT2 was responsible for flowering but not bulbing. We were unable to detct *AcFT2* in *Renate* but it was expressed in *Hojem* during the later stage of bulb development and maturity (Supplementary Fig. S5). The expression of *AcFT2* is quite similar to the expression of *AcFT1* in *Hojem* at 12 h but the functional significance of *AcFT2* expression is not known. Two-way ANOVA confirmed that the variety and days from sowing had no significant effects on *AcFT2* expression (Supplementary Table S13).

#### Expression of *AcLFY* and *GA3Ox1*

**Figure 3:**
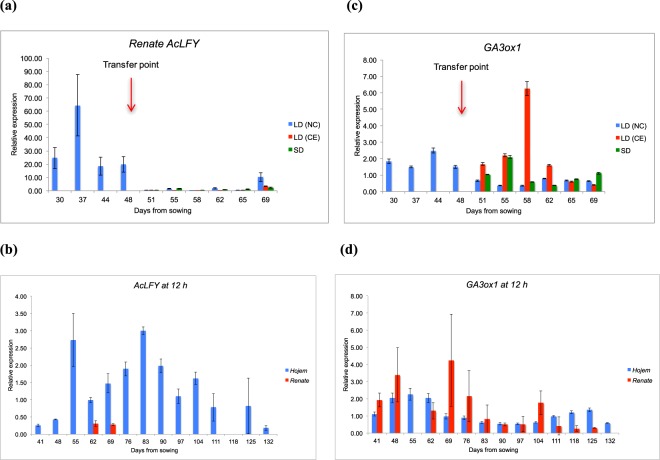
Expression of *AcLFY* and *GA3ox1* in *Renate* and *Hojem* at different daylengths. Error bars represent the SEM. (**a**) Expression of *AcLFY* in *Renate* bulb tissue at different daylengths. *AcLFY* was expressed in LD (NC) but not in LD (CE) and SD. (**b**) Expression of *AcLFY* in *Hojem* and *Renate* leaf tissue at 12 h daylight. *AcLFY* was expressed in *Hojem* throughout the development but showed very limited expression in *Renate* at the early part of the development. (**c**) Expression of *GA3ox1* in *Renate* leaf tissue at different daylengths. *GA3ox1* was expressed in all three conditions of LD (NC), LD (CE) and SD. (**d**) Expression of *GA3ox1* in *Hojem* and *Renate F1* leaf tissue at 12 h daylight. *GA3ox1* was expressed in both *Hojem* and *Renate* throughout the development.

The expression of *AcLFY* in basal scale tissues (bulb) of *Renate* was assayed by qRT-PCR during development under different daylengths. *AcLFY* was strongly expressed in basal tissue at the early stage of plant growth and at the later stage of bulb development but was not expressed in either LD (CE) or SD conditions (Fig. 3a). Early stage expression suggest that *AcLFY* might be maintaining vegetative growth in the early stage under LD (NC) but it is inhibited when bulbing starts. No expression was found in any of the three conditions after bulbing had been intiated in NC. The difference in *AcLFY* expression between LD (NC), LD (CE) and SD conditions was significant (Supplementary Table S17).

At 12 h, *AcLFY* showed significantly higher expression in *Hojem* than *Renate* throughout the early growth and development, while showing very limited expression in *Renate* at 62 and 69 DFS (Fig. 3b). As with *AcFT4*, expression of *AcLFY* in *Renate* was lower in the 12 h experiment than in Experiment 1. The level of expression of this gene was lower at the early stage of development in *Hojem*, increased during the period of bulb initiation before reducing with the bulb development and maturity. The difference in *AcLFY* expression between *Hojem* and *Renate F1* under 12 h daylength was significant (Supplementary Table S18).

In *Renate*, *GA3ox1* was expressed in all three conditions of LD (NC), LD (CE) and SD throughout the bulb initiation and development (Fig. 3c). Data suggest that *GA3ox1* was not directly involved in onion bulb initiation and development. At 12 h, *GA3ox1* was expressed throughout the growth and development in both *Hojem* and *Renate F1* (Fig. 3d). However, it was observed that the expression level was similar hroughout the growth and development in both varieties of onion.

### Spatial gene expression

An experiment was performed to determine spatial expression of the genes of interest in onion. In particular, we were interested in the expression of these genes in the site of perception (green leaf) or response (basal tissues) under bulbing and non-bulbing daylegths.

#### Expression of *FT* genes

**Figure 4:**
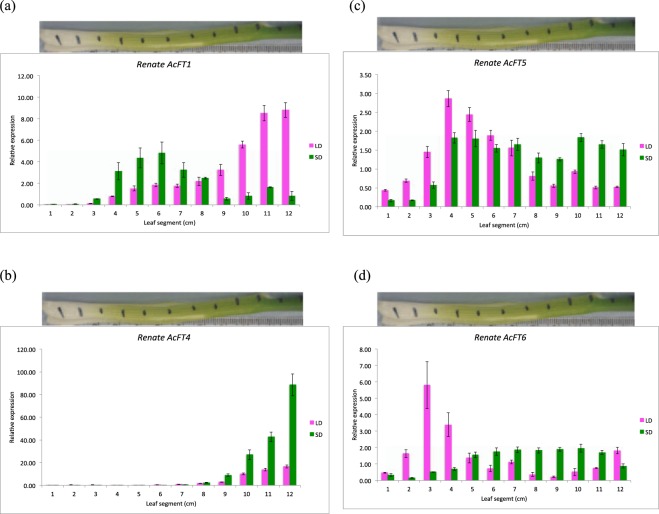
Expression of *FT* genes in *Renate* onion leaf under LD and SD conditions. Error bars represent the SEM. (**a**) Expression of *AcFT1* in onion leaf under LD and SD. *AcFT1* was expressed throughout the green part of the leaf (site of perception) but was not expressed in basal tissue (site of response) under LD and SD. (**b**) Expression of *AcFT4* in onion leaf under LD and SD. *AcFT4* was only expressed in the green part of the leaf (site of perception) but was not expressed in basal tissue (site of response) under LD and SD. (**c**) Expression of *AcFT5* in onion leaf under LD and SD. *AcFT5* was expressed all throuhout the leaf from the site of perception (green part) to the site of response (basal tissue) under LD and SD. (**d**) Expression of *AcFT6* in onion leaf under LD and SD. throughout the leaf site of perception (green part) to the site of response (basal tissue) in both LD and SD.

*AcFT1* showed differential expression in *Renate F1* leaf under LD and SD (Fig. 4a). In LD, it was expressed all throughout the green part of the leaf, although the expression was slowly increased from the transition zone to the mature green leaf tissue. In SD, the expression of *AcFT1* was limited to the transitional part of the leaf and then sharply decreased in the older green tissues at the site of perception. it was observed that in both LD and SD conditions, *AcFT1* was not expressed in the basal tissue i.e. the site of response in either LD or SD. The difference in *AcFT1* expression between LD and SD conditions was significant as was the position of the leaf segment (Supplementary Table S19).

*AcFT4* was expressed in the green part of the leaf but was not expressed in the basal tissue or transitional part of the leaf under either LD and SD conditions (Fig. 4b). However, the expression was significantly different between two daylengths, being high in SD and low in LD. This tissue-specific expression pattern of *AcFT4* in SD indicates that this gene might inhibit bulb formation in *Renate F1* at SD which is consistent with the previous experimental results. The difference in *AcFT4* expression between LD and SD and the position of the leaf segments was significant (Supplementary Table S20).

*AcFT5* was expressed throughout the leaf from the bulb through tho the oldest green tissues in both LD and SD, (Fig. 4c). However, *AcFT5* showed higher level of expression in basal tissue to the transitional part than to green leaf tissue under LD, while, it showed lower level of expresssion in the basal tissue than to the green part under SD. The difference in *AcFT5* expression between LD and SD conditions was not significant although the leaf segments had significant effect on *AcFT5* expression and the interaction between leaf segment and daylength was also shown to be significant (Supplementary Table S21). Thus, the pattern of expression of *AcFT5* the leaf segments is affected by daylength.

*AcFT6* was expressed throughout the leaf from the site of perception (green part) to the site of response (basal tissue) in both LD and SD (Fig. 4d). However, *AcFT6* showed higher level of expression in basal tissue then sharply decreased in the rest part of the leaf under LD In SD it showed lower levels of expresssion in the basal tissue and then showed similar level of expression to the rest of the leaf. ANOVA confirmed that the leaf segment position had a significant effect on *AcFT6* expression and the interaction between leaf segment and daylength was also shown to be significant (Supplementary Table S22).

#### Expression of *AcLFY* and *GA3Ox1*

**Figure 5:**
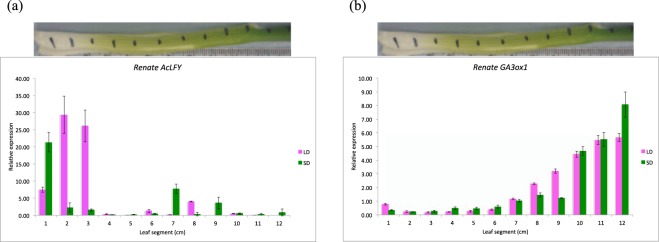
Expression of *AcLFY* and *GA3ox1* in *Renate* onion leaf under LD & SD conditions. Error bars represent the SEM. (**a**) Expression of *AcLFY* in onion leaf under LD & SD. *AcLFY* was mostly expressed in bulb tissue (site of response) under LD and SD. (**b**) Expression of *GA3ox1* in onion leaf under LD and SD. *GA3ox1* was expressed throughout the leaf under LD and SD.

*AcLFY* was mostly expressed in basal part of the leaf (site of response) under both LD and SD conditions (Fig. 5a). This result is consistent with results in the previous experiment, where *AcLFY* mRNA band was only found in basal tissue under LD and SD conditions through RT-PCR. Therefore, it could be confirmed that *AcLFY* is a bulb tissue specific gene. However, the qRT-PCR expression of *AcLFY* in *Renate F1* was significantly higher in bulb tissue under LD than to SD. Two-way ANOVA confirmed that both the difference in *AcLFY* expression between LD and SD conditions and the position of the leaf segments was significant (Supplementary Table S23). *GA3ox1* was expressed throughout the leaf under both LD and SD conditions, although the level of expression was very low in the basal tissue (site of response) and slowly increased to top of the leaf (Fig. 5b). There was no significant difference found for *GA3ox1* expression in either leaf or bulb tissue under LD and SD. This result suggests that the tissue specificity of expression of this gene is not dependent on daylength.

## Discussion

Being a biennial species, it takes more time to improve onion crops by conventional breeding methods such as hybridization, recombination and selection. Also the presence of severe inbreeding depression makes it difficult to produce and maintain a large number of near homozygous inbred lines ideal for genetic linkage and analysis^2,32,33^. Daylength sensitivity places a significant barrier to onion breeding programmes as a trait in a particular daylength group cannot be transferred to another daylength group by cross breeding because the specific daylength response of the progeny will be unknown. In addition, crossing onions with different daylength requirements is difficult, as the progeny will be compromised. In comparison to the knowledge gained regarding photoperiodic regulation of flowering, relatively little is known about genetic regulation of bulbing process and only a small number of quantitative genes with easily visible effects have been described in onion^2,9,12,34^.

The main aim of this work was to gain a better understanding of the molecular regulation of bulbing in response to daylength and specifically to test whether the molecular regulation involved genes controlling flowering by daylength in Arabidopsis. To test this hypothesis, a series of physiological and molecular experiments were carried out throughout the project. The results obtained suggest that a few genes identified and isolated in onion such as *AcFT1*, *AcFT4*, *AcFT5*, *AcFT6*, *AcLFY* and *AcGA3ox1* were potentially involved in daylength control of bulb formation and support the concept that bulb formation in response to daylength is similar to the daylength regulation of flowering in Arabidopsis.

### Daylength regulation of bulbing in onion

Bulb formation in temperate onions is daylength-dependent and LD of at least 14 h of light are required to stimulate bulb initiation^8,10,13^. Therefore, the objectives of this study were to optimise the experimental conditions for daylength-dependent bulb initiation in onion by a comprehensive set of developmental time-course experiments and to generate materials for molecular analyses. In an initial time-course experiment, it was observed that onions form bulbs (measured by bulbing ratio) around 48 DFS when daylength extends beyond 14 h. The leaf and bulb materials obtained from this experiment were used for molecular analyses. Based on the timing of the bulb response, in the later experiment in *Renate F1* shown that the bulbing ratio increased in plants kept in LD (NC) or transferred to LD (CE) at the time that bulbing was just beginning, but not in those transferred to SD at that time. Statistical analysis also supports this result. This confirmed that bulb initiation was controlled by daylength and LD conditions with fourteen to sixteen hours of light stimulating the bulbing process in *Renate F1*^16^. The onion plants grown under LD (NC) produced larger sized bulbs than those grown in LD (CE) which is probably due to the higher light levels and longer days in the glasshouse in mid summer, both of which have been shown to accelerate bulb formation^35^. The light quantities used under each daylength conditions in this experiment are shown in Supplementary Fig. S6. Light quality also has a major effect on bulb initiation^36^ with high far-red or far-red to red ratios being promotive and thought to be mediated by Phytochrome A. Blue light, to a lesser extent, also controls bulb initiation^37^. It seems that light quality has a more pronounced effect on bulb initiation in onion. Under natural conditions, light will contain both far-red and blue light and thus daylength will be the limiting factor controlling bulb initiation in LD onions^16^. This allows for a seasonal response, bulbing being initiated when the days get longer in the spring in the UK and allows for rapid bulb development under favourable environmental conditions e.g. warm temperatures and high irradiance during early to mid summer. The reason behind the delay in measurable bulb formation could be because bulb scales form quite quickly but the scales expand slowly^38^.

In ID (12 h daylength) conditions, it was observed that the SD variety *Hojem* forms bulbs at around 104 DFS, while *Renate F1* did not form any bulbs even at 132 DFS. Statistical analysis also supports this result. The daylength requirement for onion bulb formation varies with the type of cultivar, ranging from 10 to 16 hours^39^. The adaptation to a certain production area depends largely on adaptation to daylength through the daylength requirement of the specific cultivar^40^. Therefore, the daylength at a specific production area or latitude at the time of bulb initiation will influence on the selection of onion cultivars. Even though a 12 h daylength would be long enough to promote bulbing in SD varieties of onion, there was a long period of time (104 DFS) to bulb initiation in *Hojem*, compared to the time to bulbing in *Renate F1* plants grown initially in NC. This delay could be due to the reduced light integral during the early stage of plant growth in the 12 h daylength chamber compared to NC^31^. In addition, the slow increase of bulbing ratio in *Renate F1* under SD or delay in bulb initiation in *Hojem* at 12 h could be due the accumulation of an inhibitor of bulb formation in SD conditions or at constant 12 h, so that plants would then take a longer time to initiate bulbs, as the inhibitor would have to be degraded. It is not surprising that sometimes, onions sown in springtime fail to complete bulb formation and they revert to leaf blade production, resulting in thick-necked plants. It was also clearly observed that following bulb initiation in *Hojem*, bulbs grew more rapidly in plants that had bulbed later than earlier bulbing ones. This might be due to the age of plants, which has been shown to affect the rate of bulb formation^41^. Statistical analysis also showed that bulbing ratios were significantly different between *Hojem* and *Renate* varieties in response to the daylength and the age of the plants (Supplementary Table S7).

### Temporal gene expression in onion in response to daylength

Onion is a biennial plant, where bulb formation, being an overwintering stage^2^ occurs in the first year and flowering occurs following a period of vernalisation^11^ in the second year. Therefore, a question arises of which genes are involved in the photoperiodic flowering pathway and which are involved in onion bulbing^16^. It was clearly observed that onions initiate bulbing under inductive daylengths in the first year, when flowering is inhibited, suggesting that bulb formation and not floral initiation is the daylength response^2,8,12^. Previous reports also revealed that *FT* is a target of *CO*^42^ and has been shown to be the major component of the floral signal molecule, florigen and thus can induce flowering by long-distance transportation to the apical meristem with the help of other floral homeotic genes like *LFY*^19–22^. At the physiological level, bulb initiation in onion is very similar to the floral initiation in Arabidopsis^20^. Thus, FT, which is the mobile signal controlling flowering in Arabidopsis^21,43^. could control bulb initiation in onion^16^. The experimental hypothesis is that, if *FT* genes regulate bulbing, their expression should be correlated with bulb formation under a range of conditions and in different response types. For genes involved in daylength sensing, they should be related to the bulbing response e.g. present in the sites of daylength perception, but independent of the bulbing process. To test this hypothesis, this study focused on the quantitative gene expression analysis in different response types of onion under a range of bulbing and non-bulbing conditions.

#### *FT* genes

*FT* genes have been shown to act as long distance signals mediating resposnses to daylength in a range of species. In *Renate F1*, we were able to confirm the presence of six sequences as published by Lee *et al*.^9^. They proposed that *FT1* promoted bulb formation, whereas *FT4* inhibited it. We tested this hypothesis by looking at *FT* gene expression in a range of conditions. If *FT1* was correlated bulbing in response to daylength, we would expect that it would be expressed at higher levels in LD than SD in the site of perception. The data we obtained by quantitative PCR was consistent with this prediction.

During development, *FT1* was expressed in LD at all stages in *Renate*, including the early stages, prior to the onset of bulbing but not expressed in SD. In addition, *AcFT1* showed significantly higher expression in LD (NC) than in LD (CE). This could be due to the much higher light level in LD (NC) during the summer compared to the LD (CE) chamber^31^. However, transfer of plants to SD when bulbing initiated resulted in expression being repressed. Some expression of *FT1* was detected in the transition zone in SD. *FT1* is proposed to travel from the leaf to the apex as part of the supply of assimilates from photosynthesising, i.e. exporting tissues. It is likely that the transition tissue acts as a sink and does not supply assimilates to the apex. *FT1* expressed in these tissues may thus be inactive for bulbing. *AcFT1* showed very high expression in *Hojem* during the later stage of bulb development and maturity, but showed very limited, or no, expression in *Renate F1* at 12 h throughout development. This result could also be suppported by a previous study in *A. cepa*, where the authors proposed that *AcFT1* was down-regulated in the early maturity line under both SD and LD conditions (Manoharan *et al*., 2016). Overall, the expression characteristics are consistent with *FT1* promoting bulbing in onions^9^. In contrast, when *Renate F1* plants were transferred from LD to SD during development, *AcFT4* was only expressed in SD which might inhibit bulb formation but was not expressed in either LD (NC) or LD (CE) and *AcFT4* expression was upregulated at the same time that bulbing was inhibited. These results are also supported by the previous studies, where the authors proposed that *AcFT1* promotes bulb formation, while, *AcFT4* down-regulates the expression of *AcFT1*, hence inhibits bulb formation in onion^9,44^. It could be confirmed that the expression of *AcFT1* is negatively correlated with *AcFT4*.

The expression pattern in *Hojem* differed from that in *Renate F1* in that *AcFT4* was expressed during early development in ID, and decreased during bulbing, which may indicate that the function of this gene has diverged in different daylength types. The data suggest that *AcFT4* might inhibit bulb formation in *Hojem* at the early stage of growth or during the juvenile phase by suppressing *AcFT1*. In certain tree species, it was reported that the juvenile phase has been shortened by the overexpression of the *FLOWERING LOCUS T* (*FT*) gene^25^. Down-regulation of *AcFT4* in *Hojem* during bulb initiation and development could be due to the suppression by, or strong expression of, *AcFT1*. On the other hand, up-regulation of *AcFT4* might down-regulate the expression of *AcFT1* in *Renate F1* under ID conditions. Therefore, the temporal expression patterns of *FT1* and *FT4* genes in two different onion cultivars in response to daylengths suggest that these genes might be negatively co-ordinated with each other. Lee *et al*. (2013) proposed that two antagonistic FT-like genes regulate bulb initiation, where *AcFT1* promotes bulb formation, while *AcFT4* prevents upregulation of *AcFT1* and inhibits bulbing in transgenic onions^9^. These results could also be supported by the previous studies in sugar beet, where a similar regulatory pathway has evolved and was found that two *FT* genes with opposite expression profiles functions antagonistically for control of flowering^45,46^. The negative co-ordination of *FT* with *DORMANCY ASSOCIATED MADS-BOX* (*DAM*) was observed in leafy spurge (*Euphorbia esula* L.), where it was found that DAM proteins potentially controls dormancy transition and maintenance by negatively regulating the expression of *FT*^47^. *FT* also shows further inhibitory role in plants, where it controls seed dormancy through the inhibition of proanthocyanidin synthesis in fruits and thus altered the seed coat tannin content^48^.

The expression of *AcFT2* was similar to that of *AcFT1* in ID conditions. It might be speculated that both *AcFT1* and *AcFT2* induce bulb formation and development in *Hojem* although previous studies produced no evidence that *AcFT2* has a role in bulb formation but found it was a flower promoting gene^9^.

*AcFT5* showed an interesting pattern of expression in *Renate F1* under different daylengths. Like *AcFT1*, *AcFT5* showed a significantly higher level of expression in LD (NC) than in LD (CE). This could also be due to the sufficient light level in LD (NC) during the summer compared to the LD (CE) chamber^31^. *AcFT6* expression was different from that of the other *FTs* and only appeared in LD (NC). It has been demonstrated that *FT* is rapidly upregulated in Arabidopsis and other plants, when plants are shifted from non-inductive conditions to an inductive photoperiod^21,49^. *AcFT5* might not be involved in the bulb induction process itself in either *Hojem* or *Renate F1* under ID conditions. *AcFT6* was expressed at the early stage of plant growth in *Hojem*, while it was expressed at the middle stage of development in *Renate F1* at 12 h. However, these results are incosistent with the previous study of Lee *et al*. (2013), where the authors suggest that the expression of *AcFT5* and *AcFT6* were similar at the three stages (young, mature and bulb) of development and did not appear to be affected by daylength^9^. Further work is required to understand the roles of these genes.

#### *LFY* and *GA3ox1*

In this study, our hypothesis was that if *LFY* or *GA3ox1* were involved in bulb formation in response to daylength it would be reflected in their patterns of expression. In *Renate F1*, *AcLFY* was strongly expressed in bulb tissue under LD (NC) at the early stage of plant growth and at the later stage of bulb development but was not expressed in either LD (CE) or SD conditions suggested that this gene causes the plant to be less sensitive to environmental signals at this time^50^. Therefore, *AcLFY* might need *AcFT1* to correlate onion bulbing process in LD. This could be supported by the role of *LFY* in Arabidopsis, which triggers the expression of the floral homeotic genes at the floral apical meristem and causes flowering^51^, whereas, in onion, the apex is present at the base of plant (bulb). Disappearance of its expression after bulb formation in all three conditions could be due to suppression by other genes like *FLOWERING LOCUS C* (*FLC*), which is a negative repressor for autonomous pathways in Arabidopsis^20^. As there is a small increase in bulbing ratio, even in the SD treatment, it may suggest AcLFY may be involved in maintaining vegetative development. Re-emergence of *AcLFY* in LD (NC) at bulb maturity suggests that this gene might also play an important role in maintaining the apical meristem after bulb development and prior to flowering. In addition, the strong expression of *AcLFY* in *Hojem* leaf tissue under ID throughout development suggests that it might play a significant role in bulb development irrespective of onion varieties. This is supported by previous studies that reported *LFY* is expressed widely in both vegetative and reproductive tissues in a range of higher plants, and plays an important role in promoting flower formation by mutual interaction and coordination in a complex network with other genes such as *TFL, AP1, AP2, FT, AP3, CO*, and *GA1*^52,53^.

*GA3ox1*, on the other hand was expressed in *Renate F1* under all three daylengths as well as all throughout the development in both *Hojem* and *Renate F1* at 12 h, suggesting that this is daylength insensitive irrespective of onion cultivars and might not directly be involved in onion bulb formation. Gibberellin was successful to promote flowering in Arabidopsis (*A. thaliana*) through the activation of the promoter of the floral integrator gene *LFY*^54^ and is also involved in onion flowering^55^. Previous studies showed that an inhibitor of gibberellin biosynthesis promotes bulbing in non-inductive photoperiods which suggests an inhibitory role in bulb initiation^55,56^. Therefore, it can be speculated that there is a crossover between the genetic control of flowering and bulbing in onion. Although the role of GA has not been fully characterised in onion, it was clearly shown that *GA3ox1* was strongly expressed in the green part of the leaf, which is the site of perception. It may be that there are other members of the gene family in onion, one or more of which might be related to the bulb response. This could be supported by previous studies, where four *GA3ox* genes were identified in Arabidopsis, each of which exhibits a unique organ-specific expression pattern; suggesting individual *AtGA3ox* member played a distinct developmental roles^57,58^.

### Spatial gene expression in onion in response to daylength

As monocotyledon leaves develop from a basal meristem, during leaf growth, cells move through the basal region into a transition zone and then into the green, photosynthesising part of the leaf. The photosynthetic and basal parts of the leaf can be considered as the sites of perception and response, respectively. By looking at the expression of genes at different points along the length of the leaf, it is possible to determine the extent to which their expression correlates with the different functional regions and also give a picture of how expression changes in cells of increasing age. Both *AcFT1* and *AcFT4* genes showed tissue specific expression pattern under either LD or SD conditions. However, the results suggest that the both *AcFT1* and *AcFT4* genes were expressed differently under LD and SD but produced in the same leaf tissue.

It was also observed that *AcFT4* expression pattern in *Renate F1* leaf was opposite to that of *AcFT1* in both LD and SD conditions., supporting the idea that *AcFT4* is negatively correlated with *AcFT1*. *AcFT5* and *AcFT6* were expressed throughout the leaf from the site of perception (green tissue) to the site of response (basal tissue) in both LD and SD. Statistical analysis revealed that the interaction of *AcFT5* and *AcFT6* expression in leaf tissue was significantly different when grown under LD compared to that of the SD conditions, indicating that their spatial expression pattern is affected by daylength. These results could support a role for these genes in onion bulb formation but this would need to be confirmed by functional analysis of their roles.

*AcLFY* showed mostly bulb-specific expression pattern in both LD and SD conditions. Statistical analysis also supports these results. This is because *LFY* causes a group of undifferentiated cells named meristems to develop into flowers instead of leaves associated with shoots^59–67^. *GA3ox1* expression was present everywhere in the leaf tissue from the site of perception (green tissue) to the site of response (basal tissue) under both LD and SD conditions, suggesting no tissue specific expression pattern of this gene.

## Supplementary information

Supplementary information including supplementary figures and tables
