# Supplementary material for: Temporal and Spatial Expression of Arabidopsis Gene Homologs Control Daylength Adaptation and Bulb Formation in Onion (Allium cepa L.)
Source: Sci Rep. 2019 Oct 10;9:14629. doi: 10.1038/s41598-019-51262-1 (PMC6787249; doi:10.1038/s41598-019-51262-1)
Supplement: Supplementary file 1 — Supplementary information including supplementary figures and tables [file 41598_2019_51262_MOESM1_ESM.doc]

**Supplementary information**

**Title Page:**

**Title:** Temporal and Spatial Expression of Arabidopsis Gene Homologs Control Daylength Adaptation and Bulb Formation in Onion (*Allium cepa* L.)

**Running head:** *Temporal-Spatial Gene Expression of Onion Bulbing*

**Author’s name:** Md. Harun Ar Rashid[[1]](#footnote-2)*, Wei Cheng^2^, Brian Thomas^2^

**Authors’ address:**

^1^School of Life Sciences, Gibbet Hill Campus, The University of Warwick, Coventry, CV4 7AL, UK, Corresponding author email: harun_hort@bau.edu.bd

^2^School of Life Sciences, Gibbet Hill Campus, The University of Warwick, Coventry, CV4 7AL, UK

**Supplementary figures**

**Supplementary Figure S1**. Growth of *Renate F1* plants under natural condition in Phytobiology Facility to generate materials for molecular analyses. LD-grown bulbs are shown. A similar method was employed for plants grown in SD and other developmental experiments.

**Supplementary Figure S2.** Method of transfer experiment during development of onion cv*.* *Renate F1.*


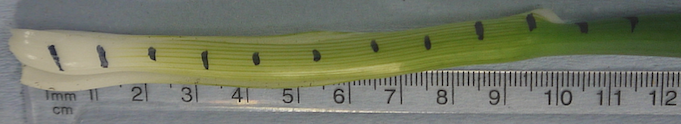


Supplementary Figure S3. Leaf sections for spatial expression experiment in *Renate F1*

| (a) | (b) |
| --- | --- |
| (c) | (d) |
| (e) | (f) |

**Supplementary Figure S4**. Gel red stained agarose gel showing amplification of different genes in onion. (a) *FT* mRNA in *Renate F1*. 1=*AcFT1*, 2=*AcFT2*, 3=*AcFT3*, 4=*AcFT4*, 5=*AcFT5*, 6)=*AcFT6*, M=marker. (b) *AcFT1* and *AcFT4* in *Renate F1*. M=marker, C= cDNA, W=water control, G=genomic DNA. (c) *AcFT2* in *Hojem* at 12 h. M=marker. (d) *AcFT2* in *Renate F1* at 12 h. M=marker. (e) *AcLFY* in *Renate F1*. M=marker. (f) *GA3ox1* in *Renate F1.* M=marker.

**Supplementary Figure S5.** Expression of *AcFT2* in *Hojem* and *Renate F1* at 12 h. *AcFT2* was expressed in *Hojem* after bulb formation but not expressed in *Renate*. Error bars represent the SEM.

**Supplementary Figure S6.** Light quantities used for growth of *Renate F1* under different daylength conditions. (NC: natural condition, LD: long day, SD: short day, CE: controlled environment, nm: nanometre).

**Supplementary tables:**

**Supplementary Table S1.** Control primers designed from onion EST sequence used to confirm the expression of genes of interest in onion. Forward is top in each section and Reverse is below.

| Gene | GeneBank Accession | Forward (RT-FOR) and Reverse (RT-REV) primer sequences (5’…..3’) | Annealing temperature (^o^C) | Product size (bp) | Gene size (bp) |
| --- | --- | --- | --- | --- | --- |
|
| *ALL* | L48614.1 | TTCAGAAACTAAGAGAGAGGT  CTTCCCATTCACACTTCACCC | 53 | 160 | 4014 |
| *FKF1* | GQ232754 | AGGTCGCAATTGCCGATTCTTA  AACGTGACCCGTTCATGGAAGT | 60 | 815 | 2054 |
| *GIa* | GQ232756 | TGGTTGCTGCACATGTTTCTGA  GTGTGCACTTGGATGCGATAGG | 60 | 956 | 3555 |
| *GIb* | GQ232757 | TGGTTGCTGCACATGTTTCTGA  GTGTGCACTTGGATGCGATAGG | 60 | 956 | 3555 |
| *LFY* | JX275963 | ACAACCCTCTCCCACCTCTTCC  TTCTTCATCCAGGCAATGCAAA | 60 | 642 | 1119 |
| *COL1* | GQ232751 | GAATCCGACGGTGCAGGTTAAG  GCCTTTCTGGAAGCGTAGCGTA | 60 | 522 | 1109 |
| *GA3ox1* | AB303422 | AACACTTCGACCTCGGATCAGC  TATGGTCAGCAGGCTTGAGTCG | 60 | 644 | 1208 |
| *FT-LIKE PROTEIN 1* | JX145040 | TTGGAGATGTTGTGGATCCGTTT  CAATGGATCAAGATGCGGTCAG | 60 | 515 | 807 |
| *FT-LIKE PROTEIN 2* | JX145039 | GAAACGTTGTGGGCGATGTTCT  GGAACGGAATGGTAGGATGCAG | 60 | 579 | 834 |

Legends: *ALL: ALLINASE, FKF1: FLAVIN-BINDING KELCH REPEAT PROTEIN; F-BOX 1 PROTEIN, GI: GIGANTEA, LFY: LEAFY, COL1: CONSTANS LIKE 1, GA3ox1: GIBBERELLIN 3-OXIDASE, FT-LIKE PROTEIN: FLOWERING LOCUS T-LIKE PROTEIN.*

**Supplementary Table S2. Control primers designed from onion EST sequence used to confirm the expression of *FT* genes in *Renate F1* (Lee et al., 2013). Forward is top in each section and Reverse is below.**

| Gene | GeneBank Accession | Forward (RT-FOR) and Reverse (RT-REV) primer sequences (5’…..3’) | Annealing temperature (^o^C) | Product size (bp) | Gene size (bp) |
| --- | --- | --- | --- | --- | --- |
| *AcFT1* | KC485348 | TTACATGGCAAGAGAAAGTGACCCAT  CCTATTAGTAATCCGTGTATATTCT | 50 | 537+8=545 | 726 |
| *AcFT2* | KC485349 | AAGGATGATGGATTCGGATCCGTTA  CCATTCATCTATAAGTTCTCCTCCCA | 61 | 528+8=534 | 572 |
| *AcFT3* | KC485350 | GGGAATGTTGTAGGCGATGTTT  AGCCGTTTCCTGGTCGATACCT | 50 | 508 | 745 |
| *AcFT4* | KC485351 | CACGATGTCTTTTGATCCTTTAGTT  TGTGCTAATTCTCTGATCGAAACCTT | 50 | 528+8=534 | 539 |
| *AcFT5* | KC485352 | TTCCATGTCAAGAGATCCTCTTGTT  AGTGTCAGAGCCAGCCACTTCCT | 58 | 540+8=548 | 604 |
| *AcFT6* | KC485353 | ATACATGCAAGTAAAAATGTTGCGA  GCAGTCAGCAAAGCCCCGAGAACCT | 58 | 558+8=566 | 841 |

Legends: *AcFT: Allium cepa FLOWERING LOCUS T*. Green colour means start codon and red colour means stop codon.

***Supplementary Table S3. qRT-PCR primers used to detect reference genes and their similarities with Renate F1 onion transcriptome sequence. Forward is top in each section and Reverse is below.***

| Gene | Onion GeneBank ID (NCBI) | Arabidopsis GeneBank ID (NCBI) | Forward (qRT-FOR) and Reverse (qRT-REV) primer sequences (5’…..3’) | Product size (bp) | Nucleotide sequence length (bp) | | Similarity between NCBI database sequence and onion transcriptome sequence | |
| --- | --- | --- | --- | --- | --- | --- | --- | --- |
|  |  |  |  |  | NCBI Reference sequence | *Renate F1* transcriptome | % Nucleotide | % Amino acid |
| *UBL* | KY072874 | NM_129118.4 | CTGTCCTTCATCTTGTGCTTGCTCT  CAAGACCGAAACAAACCATCAAATTA | 148 | 712 | 596 | 84 | 94.8 |
| *TIP-41* | KY072882 | NM_119592.4 | GTCAAACACAGAACTGAAAGGCTGAA  AATAAATGATTGTTGGCTCGCATCTA | 146 | 1254 | 1456 | 71 | 66.5 |
| *PP2AA3* | KY072881 | BT002601.1 | ATCGATAAAGCCGTGTCTAGTTGAGC  GCGACAAATCATGATACTTTCTGACA | 134 | 1794 | 2065 | 77 | 84 |
| *PP2A-1* | KY072880 | AY096543.1 | CAGATACAACACGCAAGACTCCTGAT  CCCACTCCCTTTGTAGCACAAATCT | 127 | 952 | 1518 | 79 | 90.2 |

Legends: *UBL: UBIQUITIN-LIKE PROTEIN RUB2, TIP-41: TONOPLASTIC INTRINSIC PROTEIN-41, PP2AA3: PROTEIN PHOSPHATASE 2A REGULATORY SUBUNIT 3, PP2A-1: PROTEIN PHOSPHATASE TYPE 2A.*

**Supplementary Table S4.** Primers for qRT-PCR used to estimate the expression of genes of interest in onion. Forward is top in each section and Reverse is below.

| Gene | GeneBank  Accession | Forward (qRT-FOR) and Reverse (qRT-REV) primer sequences (5’…..3’) | Annealing temperature (^o^C) | Product size (bp) | Primer concentration for qPCR (µM) |
| --- | --- | --- | --- | --- | --- |
| *AcFKF1*  (2054 bp) | GQ232754 | TTTGGCATATTGGGTACTGTAGGTA  ACGAGCACAATCAGATTTATACAACAGC | 60 | 130 | 0.3 |
| *AcGI*  (3555 bp) | GQ232756 | CACAGATGGATTGCTTGTTGATG  ATTGGCTACGAGATGAACTGCTC | 61 | 94 | 0.3 |
| *AcCOL1*  (1109 bp) | GQ232751 | AGAGAAGCGAAAGAATAGAAAGTT  ATCCGCATAAGAATCGTTGTC | 55 | 127 | 0.2 |
| *AcCOL2*  (1323 bp) | KY012331 | ATTGATCAGGTGCTGAAGGGATTG  AATCGTCACCATTAAACTACACTGAAA | 65 | 143 | 0.2 |
| *AcCOL3*  (1623 bp) | KY012332 | CGAAGTTGAGTTTCACCGTGTACTTG  TGCTCTGGTACTGAGCATACAACAAA | 60 | 135 | 0.2 |
| *AcFT1*  (726bp) | KC485348 | AAACCATCACAAATAACTCAGCA  GTTTCTCGCCCAAAGTTCG | 56 | 185 | 0.2 |
| *AcFT2*  (572bp) | KC485349 | AAGTTGCTAATGGACGCGAGTTTAAG  CACCAACACAAGTGCATAAGAGTTCC | 61 | 104 | 0.2 |
| *AcFT3*  (745bp) | KC485350 | AGGAAGTTACTAACGGGTGTGAA  CAAAGCTTGCATCTTTTGACC | 60 | 201 | 0.2 |
| *AcFT4*  (539bp) | KC485351 | TGAAATAGGAGGTGTACCAAGAAT  TTCCGAAACTACCATCCATATTTG | 60 | 143 | 0.3 |
| *AcFT5*  (604bp) | KC485352 | GAAATTGGAGGACGCGAC  CTTGCATCTTTTGCTTCTGGTA | 60 | 137 | 0.2 |
| *AcFT6*  (841bp) | KC485353 | TCGTCAATCGATGGTTATAAATCA  TTTCCATAACTTGCATCGACTGT | 60 | 180 | 0.2 |
| *AcLFY*  (1119 bp) | JX275963 | AGCGTGCTATCAACCGATAGTAGTGA  AGCTTAGTCGGAACATACCAAATGGA | 60 | 108 | 0.3 |
| *GA3ox1*  (1208 bp) | AB303422 | GCTATTTGACAAAGCCCTAGCATCTG  ATCATACGCAACTAAGCAAGCATGTG | 63 | 86 | 0.3 |

Legends: *AcFT: A. cepa FLOWERING LOCUS T, AcFKF1: A. cepa FLAVIN-BINDING KELCH REPEAT; F-BOX 1 PROTEIN, AcGI: A. cepa GIGANTEA, AcCOL: A. cepa CONSTANS LIKE, GA3ox1: GIBBERELLIN 3-OXIDASE 1, AcLFY: A. ceap LEAFY.*

**Supplementary Table S5**. The thermal cycling condition for qRT-PCR

| **qRT-PCR protocol** |
| --- |
| 1: 95.0°C for 2:00 min  2: 95.0°C for 15 sec  3: X°C for 1:00 min (*Annealing temperature will be variable depending on the gene of interest*)  Plate Read  4: GOTO 2, 39 more times  5: 95.0°C for 10 sec  6: Melt Curve 60.0°C to 95.0°C: Increment 0.5°C 5 sec |

**Supplementary Table S6. Assessment of the significance of differences in bulbing ratio in *Renate* between daylength treatments and their interactions with DFS.**

| Sources of Variation | Sum of squares | Degrees of Freedom (df) | Mean Square | F | Significance at 95% confidence interval |
| --- | --- | --- | --- | --- | --- |
| Daylength | 9.143 | 2 | 4.572 | 32.174 | <0.05 |
| Days from sowing | 40.670 | 9 | 4.519 | 38.397 | <0.05 |
| Daylength x  Days from sowing | 8.537 | 18 | 0.474 | 4.030 | <0.05 |
| Error | 6.355 | 54 | 0.118 |  |  |

**Supplementary Table S7.** Assessment of the significance of differences in bulbing ratio at 12 h between variety (*Renate* & *Hojem*) treatments and their interactions with DFS.

| Sources of Variation | Sum of squares | df | Mean Square | F | Significance at 95% confidence interval | Partial Eta Squared |
| --- | --- | --- | --- | --- | --- | --- |
| Variety | 4.23 | 1 | 4.523 | 720.70 | <0.05 | 0.994 |
| Days from sowing | 14.22 | 13 | 1.09 | 100.08 | <0.05 | 0.962 |
| Variety x  Days from sowing | 3.45 | 13 | 0.27 | 24.29 | <0.05 | 0.859 |
| Error | 0.57 | 52 | 0.011 |  |  |  |

**Supplementary Table S8.** Analysis of Variance table showing the significance of the differences in *AcFT1* expression in *Renate F1* between treatments

| Sources of Variation | Sum of squares | Degrees of Freedom (df) | Mean Square | F-Statistic (F) value | Probability (P) value |
| --- | --- | --- | --- | --- | --- |
| Daylength | 395.6 | 2 | 197.8 | 485.1 | <0.05 |
| Days from sowing | 3.91 | 5 | 0.782 | 1.918 | >0.05 |
| Daylength x  Days from sowing | 16.22 | 10 | 1.622 | 3.978 | <0.05 |
| Error | 14.68 | 36 | 0.408 |  |  |

**Supplementary Table S9. Analysis of Variance table showing the significance of the differences in *AcFT4* expression in *Renate F1* between treatments**

| Sources of Variation | Sum of squares | Degrees of Freedom (df) | Mean Square | F-Statistic (F) value | Probability (P) value |
| --- | --- | --- | --- | --- | --- |
| Daylength | 439331 | 2 | 219665 | 188.7 | <0.05 |
| Days from sowing | 174085 | 5 | 34817 | 29.92 | <0.05 |
| Daylength x  Days from sowing | 331990 | 10 | 33199 | 28.53 | <0.05 |
| Error | 41899 | 36 | 1164 |  |  |

**Supplementary Table S10. Analysis of Variance table showing the significance of the differences in *AcFT5* expression in *Renate F1* between treatments**

| Sources of Variation | Sum of squares | Degrees of Freedom (df) | Mean Square | F-Statistic (F) value | Probability (P) value |
| --- | --- | --- | --- | --- | --- |
| Daylength | 388.2 | 2 | 194.1 | 212.3 | <0.05 |
| Days from sowing | 117.9 | 5 | 23.58 | 25.79 | <0.05 |
| Daylength x  Days from sowing | 224.7 | 10 | 22.47 | 24.58 | <0.05 |
| Error | 32.91 | 36 | 0.914 |  |  |

**Supplementary Table S11**. Analysis of Variance table showing the significance of the differences in *AcFT6* expression in *Renate F1* between treatments

| Sources of Variation | Sum of squares | Degrees of Freedom (df) | Mean Square | F-Statistic (F) value | Probability (P) value |
| --- | --- | --- | --- | --- | --- |
| Daylength | 20823 | 2 | 10411 | 25.34 | <0.05 |
| Days from sowing | 7843 | 5 | 1569 | 3.817 | <0.05 |
| Daylength x  Days from sowing | 12201 | 10 | 1220 | 2.969 | <0.05 |
| Error | 14793 | 36 | 410.9 |  |  |

**Supplementary Table S12.** Analysis of Variance table showing the significance of the differences in *AcFT1* expression at 12 h between treatments

| Sources of Variation | Sum of squares | Degrees of Freedom (df) | Mean Square | F-Statistic (F) value | Probability (P) value |
| --- | --- | --- | --- | --- | --- |
| Variety | 403.1 | 1 | 403.1 | 4.026 | <0.05 |
| Days from sowing | 2877 | 13 | 221.3 | 2.211 | <0.05 |
| Variety x  Days from sowing | 2761 | 13 | 212.4 | 2.122 | <0.05 |
| Error | 7007 | 56 | 100.1 |  |  |

**Supplementary Table S13.** Analysis of Variance table showing the significance of the differences in *AcFT2* expression at 12 h between treatments

| Sources of Variation | Sum of squares | Degrees of Freedom (df) | Mean Square | F-Statistic (F) value | Probability (P) value |
| --- | --- | --- | --- | --- | --- |
| Variety | 512 | 1 | 512 | 3.35 | >0.05 |
| Days from sowing | 3446 | 13 | 265 | 1.734 | >0.05 |
| Variety x  Days from sowing | 3661 | 13 | 281.6 | 1.843 | >0.05 |
| Error | 8558 | 56 | 152.8 |  |  |

**Supplementary Table S14.** Analysis of Variance table showing the significance of the differences in *AcFT4* expression at 12 h between treatments

| Sources of Variation | Sum of squares | Degrees of Freedom (df) | Mean Square | F-Statistic (F) value | Probability (P) value |
| --- | --- | --- | --- | --- | --- |
| Variety | 53.61 | 1 | 53.61 | 35.36 | <0.05 |
| Days from sowing | 205 | 13 | 15.77 | 10.4 | <0.05 |
| Variety x  Days from sowing | 260 | 13 | 20 | 13.19 | <0.05 |
| Error | 84.91 | 56 | 1.516 |  |  |

**Supplementary Table S15.** Analysis of Variance table showing the significance of the differences in *AcFT5* expression at 12 h between treatments

| Sources of Variation | Sum of squares | Degrees of Freedom (df) | Mean Square | F-Statistic (F) value | Probability (P) value |
| --- | --- | --- | --- | --- | --- |
| Variety | 0.415 | 1 | 0.415 | 0.432 | >0.05 |
| Days from sowing | 33.51 | 13 | 2.577 | 2.679 | <0.05 |
| Variety x  Days from sowing | 42.58 | 13 | 3.276 | 3.405 | <0.05 |
| Error | 53.87 | 56 | 0.962 |  |  |

**Supplementary Table S16**. Analysis of Variance table showing the significance of the differences in *AcFT6* expression at 12 h between treatments

| Sources of Variation | Sum of squares | Degrees of Freedom (df) | Mean Square | F-Statistic (F) value | Probability (P) value |
| --- | --- | --- | --- | --- | --- |
| Variety | 0.806 | 1 | 0.806 | 0.214 | >0.05 |
| Days from sowing | 509.4 | 13 | 39.18 | 10.44 | <0.05 |
| Variety x  Days from sowing | 814 | 13 | 62.62 | 16.68 | <0.05 |
| Error | 210.2 | 56 | 3.754 |  |  |

**Supplementary Table S17. Analysis of Variance table showing the significance of the differences in *AcLFY* expression in *Renate F1* between treatments**

| Sources of Variation | Sum of squares | Degrees of Freedom (df) | Mean Square | F-Statistic (F) value | Probability (P) value |
| --- | --- | --- | --- | --- | --- |
| Daylength | 24.58 | 2 | 12.29 | 6.355 | <0.05 |
| Days from sowing | 171.8 | 5 | 34.35 | 17.76 | <0.05 |
| Daylength x  Days from sowing | 92.98 | 10 | 9.298 | 4.808 | <0.05 |
| Error | 69.63 | 36 | 1.934 |  |  |

**Supplementary Table S18**. Analysis of Variance table showing the significance of the differences in *AcLFY* expression at 12 h between treatments

| Sources of Variation | Sum of squares | Degrees of Freedom (df) | Mean Square | F-Statistic (F) value | Probability (P) value |
| --- | --- | --- | --- | --- | --- |
| Variety | 29.77 | 1 | 1.317 | 166.2 | <0.05 |
| Days from sowing | 17.07 | 13 | 1.313 | 7.334 | <0.05 |
| Variety x  Days from sowing | 17.12 | 13 | 29.77 | 7.357 | <0.05 |
| Error | 10.03 | 56 | 0.179 |  |  |

**Supplementary Table S19. ANOVA table showing the significance of the differences in spatial expression of *AcFT1* in *Renate F1* leaf between treatments**

| Sources of Variation | Sum of squares | Degrees of Freedom (df) | Mean Square | F-Statistic (F) value | Probability (P) value |
| --- | --- | --- | --- | --- | --- |
| Daylength | 17.75 | 1 | 17.75 | 28.91 | <0.05 |
| Days from sowing | 184.1 | 11 | 16.74 | 27.26 | <0.05 |
| Daylength x  Days from sowing | 232 | 11 | 21.09 | 34.35 | <0.05 |
| Error | 29.47 | 48 | 0.614 |  |  |

**Supplementary Table S20. ANOVA table showing the significance of the differences in spatial expression of *AcFT4* in *Renate F1* leaf between treatments**

| Sources of Variation | Sum of squares | Degrees of Freedom (df) | Mean Square | F-Statistic (F) value | Probability (P) value |
| --- | --- | --- | --- | --- | --- |
| Daylength | 1890 | 1 | 1890 | 110.5 | <0.05 |
| Days from sowing | 17729 | 11 | 1612 | 94.24 | <0.05 |
| Daylength x  Days from sowing | 7622 | 11 | 692.9 | 110.5 | <0.05 |
| Error | 820.9 | 48 | 17.1 |  |  |

**Supplementary Table S21. ANOVA table showing the significance of the differences in spatial expression of *AcFT5* in *Renate F1* leaf between treatments**

| Sources of Variation | Sum of squares | Degrees of Freedom (df) | Mean Square | F-Statistic (F) value | Probability (P) value |
| --- | --- | --- | --- | --- | --- |
| Daylength | 0.048 | 1 | 0.048 | 1.046 | >0.05 |
| Days from sowing | 25.21 | 11 | 2.292 | 50.44 | <0.05 |
| Daylength x  Days from sowing | 9.747 | 11 | 0.886 | 19.5 | <0.05 |
| Error | 2.181 | 48 | 0.045 |  |  |

**Supplementary Table S22. ANOVA table showing the significance of the differences in spatial expression of *AcFT6* in *Renate F1* leaf between treatments**

| Sources of Variation | Sum of squares | Degrees of Freedom (df) | Mean Square | F-Statistic (F) value | Probability (P) value |
| --- | --- | --- | --- | --- | --- |
| Daylength | 1.163 | 1 | 1.163 | 2.946 | >0.05 |
| Days from sowing | 30.47 | 11 | 2.77 | 7.019 | <0.05 |
| Daylength x  Days from sowing | 70.94 | 11 | 6.449 | 16.34 | <0.05 |
| Error | 18.95 | 48 | 0.395 |  |  |

**Supplementary Table S23. ANOVA table showing the significance of the differences in spatial expression of *AcLFY* in *Renate F1* leaf between treatments**

| Sources of Variation | Sum of squares | Degrees of Freedom (df) | Mean Square | F-Statistic (F) value | Probability (P) value |
| --- | --- | --- | --- | --- | --- |
| Daylength | 129.3 | 1 | 129.3 | 15.32 | <0.05 |
| Days from sowing | 2939 | 11 | 267.2 | 31.66 | <0.05 |
| Daylength x  Days from sowing | 2637 | 11 | 239.8 | 28.41 | <0.05 |
| Error | 506.3 | 48 | 8.439 |  |  |

1. The nucleotide sequences reported in this paper have been submitted to NCBI database (NCBI, 2016) with accession numbers (KY072880, KY072874, KY072882, KY072881, JX275963, AB303422).

   ^1^Present address of the corresponding author: Dr. Md. Harun Ar Rashid, Associate Professor, Department of Horticulture, Bangladesh Agricultural University, Mymensingh-2202, Bangladesh, Email: harun_hort@bau.edu.bd Tel: +8801774573174, Fax: +8809161510 [↑](#footnote-ref-2)
